# Promoting continuous rehabilitation with AI-based motion capture and biomechanical analysis: objective feedback as a catalyst

**DOI:** 10.3389/fresc.2025.1697690

**Published:** 2026-02-13

**Authors:** Kotaro Matsuura, Akiko Matsuura, Miwa Izaki, Shigekazu Ishihara, Keiko Ishihara, Kosuke Morinaga, Yuichi Kurita

**Affiliations:** 1 Graduate School of Advanced Science and Engineering, Hiroshima University, Higashi-Hiroshima, Japan; 2 e-exercise Co., Ltd., Nagasaki, Japan; 3 Izaki Neurosurgery and Internal Medicine, Nagasaki, Nagasaki, Japan; 4 Department of Psychology, Hiroshima International University, Hiroshima, Hiroshima, Japan; 5 Department of Sociology, Hiroshima International University, Hiroshima, Hiroshima, Japan; 6 Department of Rehabilitation, Faculty of Rehabilitation, Hiroshima International University, Hiroshima, Hiroshima, Japan

**Keywords:** AI-based motion capture, biomechanical analysis, objective feedback, continuous rehabilitation, rehabilitation exercises

## Abstract

**Introduction:**

Exercise therapy is effective for various diseases, but one of the problems in rehabilitation is “continuity” and preventing quitting. This study assumes that objectively tracking changes in motor skills and providing feedback may contribute to the continuity of rehabilitation. Upper limb joint moments were estimated by our program using coordinates obtained through AI-based markerless motion capture. This study aimed to visualize and provide feedback on participants’ assessments of their motor function to promote an understanding of changes in their motor function and to improve their motivation to participate in the exercise. Then, the promoting effect of the feedback is examined with a questionnaire.

**Methods:**

Participants were late-stage elderly (*n* = 31) patients of a hospital's rehabilitation facilities. The four disease groups included Cerebrovascular Disease, Parkinson's Disease, Diabetes mellitus, and walking problems with Hallux Valgus. The experiment was done with a non-random assignment design. They were divided into two groups: with feedback (intervention group) and without feedback (control group), by balancing the number of participants for each disease. The intervention period consisted of 8 weeks of exercise, with a feedback report provided after 4 weeks and a follow-up after an additional 4 weeks. The original questionnaire (MEQ: Motivation in Exercise Questionnaire), comprising three questions, was used to assess motivation for exercising, self-understanding of improvements in athletic ability, and intention of continuing exercising. MEQ was administered at the start of the program and after each feedback. Changes in questionnaire scores were examined by multiple regression analysis, with explanatory variables of the kind of disease and with or without feedback.

**Results:**

The t-test was performed on each partial regression coefficient, and Parkinsonism participants had significantly negative values compared to those in other disease groups (*t*(26) = −2.43, *p* = 0.022, 95%CI [−1.85, −0.15]). For participants other than those with Parkinson's disease, changes in questionnaire scores were analyzed using a paired t-test and Wilcoxon signed-rank test. There was a tendency for scores to improve in the feedback group regarding willingness and to continue to participate in exercising. Q1 “Did participating in the exercise motivate you to participate in the exercise?” (one-sided, difference = 0.72, *t*(10) = 1.39, *p* = 0.09, 95%CI [−0.22, inf], Cohen's *d* = 0.50; 95%CI [−0.36, 1.34]). Q3 regarding motivation to continue exercising (“Do you want to continue exercising?”, (one-sided, mean difference = 0.64, *t*(10) = 1.47, *p* = 0.09, *d* = 0.43; 95%CI [−0.42, 1.27]). Q2 “Were you able to understand changes in your athletic ability?” has a smaller difference (one-sided, mean difference = 0.55, *t*(10) = 1.20, *p* = 0.13, 95%CI [−0.28, inf], Wilcoxon Signed rank test: one-sided, V = 25.5, *p* = 0.15, *d* = 0.38; 95%CI [−0.47, 1.22]).

**Discussion:**

As indicated by the changes in the scores of the questionnaire survey in this study, the participants' motivation to participate improved after the feedback was given; however, these positive effects had disappeared in the measurement one month later. Thus, continuous feedback more than once per month seems to be required to keep motivation to exercise.

## 1 Introduction

Preventing the decline in activities of daily living (ADL) function and maintaining and improving ADL function are essential for enhancing the quality of life (QOL) in “whole-person restoration,” which is interpreted broadly as rehabilitation. Rehabilitation is vital in Japan, where the aging population and various medical professionals support it.

Japan is a super-aging society unlike any other in the world, with the aging rate steadily increasing since then, and the rate was projected to reach 28.7% by March 2020 [Ministry of Internal Affairs and Communications, 2021]. According to an international comparison of the time it takes to transition from an aging society with an aging rate of 7% to a super-aging society with an aging rate of 14%, Japan achieved this milestone in only 24 years, compared to 114 years in France, 82 years in Sweden, 72 years in the United States, 46 years in the United Kingdom, and 42 years in Germany. These statistics underscore how rapidly the aging of society is progressing in Japan. According to the White Paper on Aging (Cabinet Office, Government of Japan), average and healthy life expectancy have increased. However, the increase in healthy life expectancy has been comparatively small [Cabinet Office Japan, 2021]. Exercise therapy aimed at preventing and maintaining or improving decline in ADL function effectively extends healthy life expectancy and is widely practiced. Several studies have supported that exercise therapy in older people is beneficial for the prevention and treatment of type II diabetes mellitus, mild cognitive impairment, and various lifestyle-related diseases associated with insulin resistance (1). Beyond Japan, several prominent organizations, including the American College of Sports Medicine, American Heart Association, American Academy of Family Physicians, American College of Obstetricians and Gynecologists, and the US Surgeon General's Office, have endorsed the promotion of exercise among older adults.

Rehabilitation and exercise therapy are effective and recommended for various conditions, including cardiovascular disease, diabetes, dementia, sarcopenia, and metabolic diseases (2). Mok et al. (3) suggested an inverse association between regular physical activity and all types of mortality, including cancer and vascular disease, in a general British population follow-up study. The importance of daily physical activity has recently become increasingly recognized in managing vascular diseases, particularly in the treatment process of patients with acute myocardial infarction (4). Recommendations by the American Heart Association also suggest that increasing the amount of daily physical activity may increase physical fitness, including general endurance and muscle strength, and reduce the risk of coronary artery disease. Shari et al. suggested that physical activity may delay the onset and progression of type 2 diabetes and its cardiovascular sequelae through positive effects on body weight, insulin sensitivity, glycemic control, blood pressure, lipid profile, fibrinolysis, endothelial function, and inflammatory defense systems (25). A meta-analysis by Teasel et al. analyzed RCTs in which rehabilitation intervention was initiated 6 months after the onset of cerebrovascular disease. It revealed that the majority of RCTs showed a positive effect. This study provides solid evidence of the need for rehabilitation interventions in the chronic phase of cerebrovascular disease (5). A survey conducted by the Centers for Disease Control and Prevention, the American College of Sports Medicine (CDC/ACSM), and the American Heart Association (AHA) focused on older adults with low levels of physical fitness. Sun et al. (6) suggested that for older adults with a certain level of physical fitness, increasing daily physical activity is less likely to cause a decline in physical fitness than doing nothing.

The effectiveness of rehabilitation in older adults with low fitness, the elderly with a general level of physical fitness, and patients who have completed acute rehabilitation for an extended period suggests that long-term intervention is beneficial and should not be interrupted. A significant barrier to sustaining long-term exercise interventions is the self-judged discontinuation of participation by exercise participants themselves, particularly among elderly individuals who lack the habit of regular exercise (7). Meta-analysis by Klein et al. (1) also mentioned difficulties in the continuation of physical exercise in diabetes self-management education. One factor in this phenomenon is the participants' difficulty feeling the effects of the exercise and the changes in their bodies.

Bandura (8) found that exercise self-efficacy—confidence in one's ability to achieve and accomplish physical or cognitive exercise goals—is also a key motivator for exercise, as increased physical exercise self-efficacy and cognitive exercise motivation in older adults are directly related.

In recent years, motivation has been recognized as a multifaceted construct that cannot be validly defined or evaluated as a single entity. It arises from the complex interaction of various factors, including goals, values, self-determination, self-efficacy, and social relationships.

According to a study investigating the motivation of hospitalized patients toward rehabilitation, three key factors were identified as crucial: realization of recovery, goal setting, and practice related to the patient's experience and lifestyle (9). These three factors were also endorsed by clinicians, emphasizing their significance as motivational components in patient-centered rehabilitation.

Based on the above findings, we believe that promoting an “understanding of the effects of exercise” is effective in forming exercise habits, that is, in sustaining exercise participation, regardless of the exercise habits of individual elderly persons or their level of physical fitness. In this study, we hypothesized that a lack of understanding and realization of the effects of exercise might cause long-term withdrawal from exercise intervention, and that it would be possible to encourage exercise participation by presenting changes in these factors and making the participants realize the effects of exercise. The application of AI-based motion capture in medical and rehabilitation settings holds significant potential to enhance diagnostics, treatment, and healthcare services. While numerous studies have been conducted on AI-driven skeletal estimation, including accuracy validation, there is a noticeable scarcity of research examining its practical application in exercise therapy settings (10).

This study aimed to visualize and provide feedback on the exercise participants' assessments of their motor function to promote understanding of changes in their motor function and to improve their motivation to participate in the exercise. As a feedback method, joint moments were estimated simply and validly by markerless motion capture to measure changes in participants' exercise capacity objectively. This study was conducted as an exploratory case study with nonrandom assignment of small subject groups, aimed at examining the effects of providing motion analysis feedback on motivation for exercise participation.

## 2 Materials and methods

We conducted this research based on the idea that biomechanical analysis is an effective method for participants to recognize changes in themselves during group exercise and rehabilitation.

### 2.1 Targeted exercise program

The exercise program analyzed in this study was “e-Exercise®,” with an intensity of 3 METs. This program incorporated three components of exercise: stretching, aerobic exercise, and strength training, to maintain and improve ADL and QOL. The program was performed in the sitting position to reduce the burden on the upper and lower limbs and was designed so that even those with joint disorders could perform it efficiently. It was also programmed for post-stroke rehabilitation. This type of preventive exercise is very effective in preventing the development of diseases due to aging and the need for nursing care due to sarcopenia. In this study, we measured adduction or abduction exercises in the shoulder joint in an end-sitting position to increase the ROM (Range of Motion) of the shoulder joint and to promote awareness of stable control of the shoulder joint in an exercise program.

The shoulder joint was used as the measurement site. Notably, shoulder joint function is closely related to ADL, as is walking ability. The Japanese Orthopaedic Association has developed a comprehensive evaluation method for shoulder disorders, including pain, function (muscle strength, endurance, and ADL), joint range of motion (ROM), initial radiographs, and joint stability. Postoperative changes, changes after intra-articular injections, and changes over time are the criteria used to evaluate the efficacy of physical therapy. According to the same criteria, there are items for ADL such as “being able to open and close a sliding door” and “wearing a jacket,” indicating that it is essential to evaluate not only the ROM of the subject's joints but also the strength and endurance of the muscles. When evaluating ROM in the shoulder joint simply and objectively, compensatory movements often occur due to the complexity of the shoulder joint. Visual evaluation requires skilled experience, and especially in group exercise, evaluation can be time-consuming and subject to errors between individuals.

### 2.2 Biomechanical analysis with AI-based motion capture

Marker-based motion capture systems are the gold standard for objectively assessing human movement. However, traditional marker-based systems are expensive and are typically limited to controlled laboratory environments, making their implementation in clinical settings challenging.

To address these limitations, markerless motion capture systems utilizing computer vision and AI technology have gained significant attention. According to a comparative study by Drazan et al. (11), recent advancements in markerless motion capture have demonstrated sufficient accuracy for clinical applications, particularly in estimating joint angles. Furthermore, markerless motion capture systems offer the advantage of continuous improvement in accuracy through the enhancement of trained models and the advancement of video camera technology, highlighting their potential for widespread clinical use.

**Figure 1:**
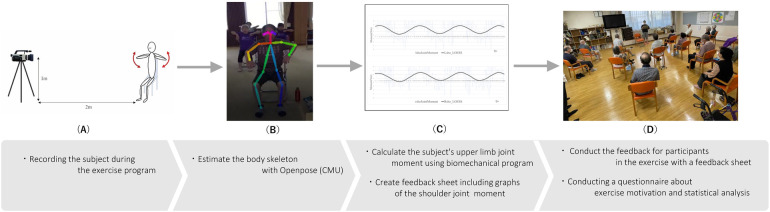
Shoulder joint moment analysis system flow: **(A)** recording of a subject in exercise. **(B)** Skeleton image of a subject estimated using OpenPose [System 1] **(C)** Analysis results of joint coordinate position changes estimated for each frame of the movie, upper limb joint torque estimated from the subject's biological data, body part inertia coefficient, and biomechanical model [System 2]. **(D)** Conducted feedback on the analysis results for exercise participants. The questionnaire about exercise motivation was carried out for statistical analysis.

The biomechanical analysis system we have developed for this study is described below. The flow of data measurement and processing required for the analysis is shown in Figure 1. The system consisted of the following: 1. a video camera to capture the subject's movement. System 1 used a video camera to capture the subject in motion (Figure 1A) and estimated the joint coordinate positions using OpenPose (Figure 1B). System 2 estimates joint moments from the joint movement.

System 1: Human skeleton estimation program utilizing OpenPose (12) estimates the coordinate positions of body feature points necessary for joint motion analysis. This program is capable of estimating a person's skeleton from a 2D video. Another advantage is its ability to process multiple persons simultaneously. It was used only to estimate joint coordinates, necessary for analyzing the movements of older adults participating in exercise. OpenPose can estimate the poses of multiple people in a single inference, using a network that predicts the possibility of joint connections, called PAFs (Part Affinity Fields), as a directional vector map. OpenPose could estimate both upper limb and lower limb joints. In an accuracy-validation experiment on OpenPose conducted by Nakano et al. (13), the system was compared with a conventional marker-based motion-capture setup. The results indicated that the mean absolute error (MAE) for joint-position estimation was below 40 mm in 80% of observations. Moreover, because performance depended on camera image quality and shutter speed, the study suggested scope for further improvements in accuracy. We apply coordinates of major joints of the upper limb (including the shoulder, elbow, and wrist) to joint moment estimation in System 2.

System 2: From the coordinates of the upper limb joints, our system estimates joint moments from angular accelerations. The joint moments facilitate the assessment of shoulder joint control stability and symmetry. Estimation computation is based on a biomechanical model (26). From the joint moments, feedback reports are made for each subject (Figure 1C).

**Figure 2:**
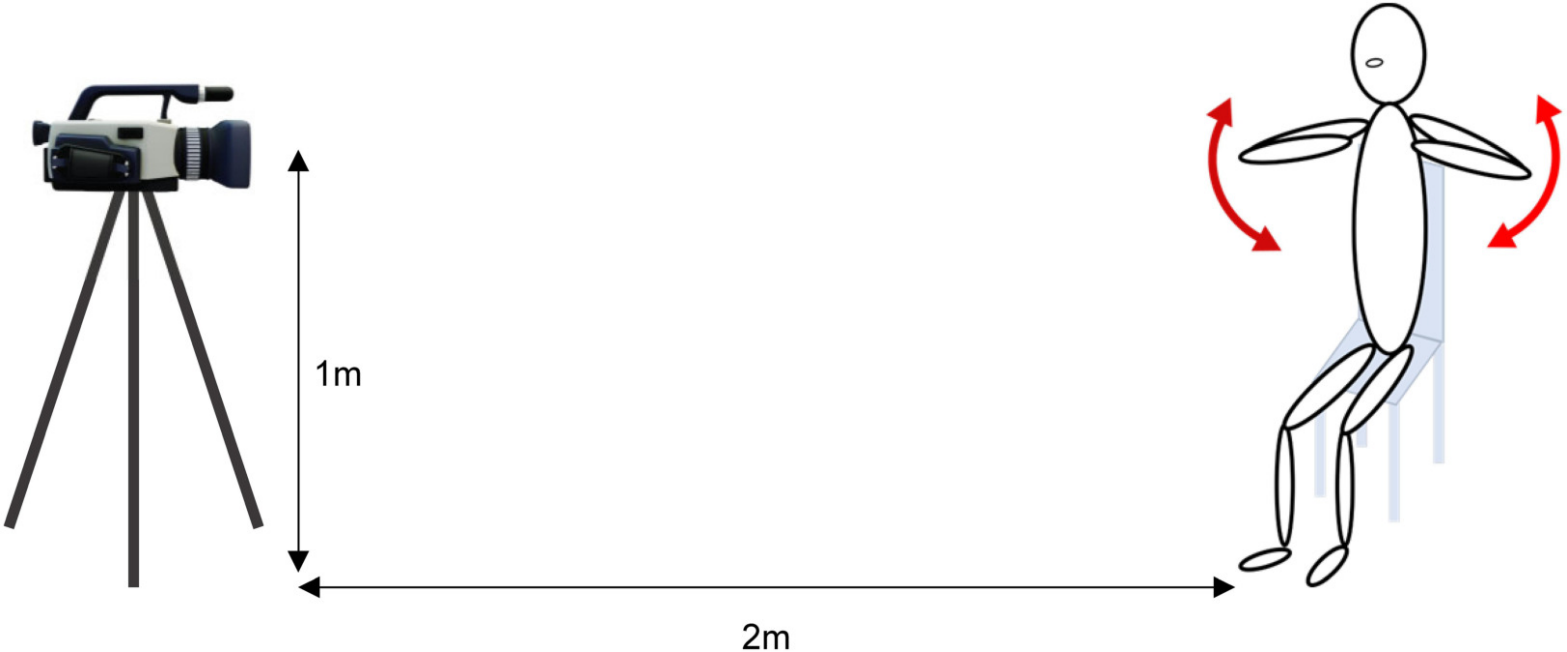
Video recording environment during exercise: the camera was positioned at a distance of 2 m from the subject and a height of 1 m from the ground. The camera captured images in full HD quality (1,080  ×  1,920) at a sampling frequency of 30 frames per second (fps).

The movement analyzed was the simultaneous adduction and abduction of both shoulder joints while sitting on a chair, and was included in the exercise program in which the subject participated(Figure 2). The x- and y-coordinates of the joint coordinates estimated using OpenPose are output as independent JSON files for each frame. This study used the joint position coordinates of the shoulder, elbow, and hand joints of both upper limbs. A program was created to estimate the upper limb joint torques from the aforementioned body dynamics model, the estimated joint coordinates, and the subject's physical information using the Scilab 5.2.2 open numerical calculation software.

Functions that provide proximal joint forces and force moments were obtained using body segment parameters, kinematic data, and distal joint forces and force moments as inputs. These mechanistic estimates can be found in previous studies (14, 15).

The equation of motion of an object in one direction is

$$F_{1}=m_{1}a_{1}$$ (1)

*m*_1_ is the body mass, and *a*_1_ is the acceleration.

The formula for finding the moment is

$$M_{1}=I_{1}a_{1f}$$ (2)

The *M_1_* is the moment, *and I_1_* is the moment of inertia.

**Figure 3:**
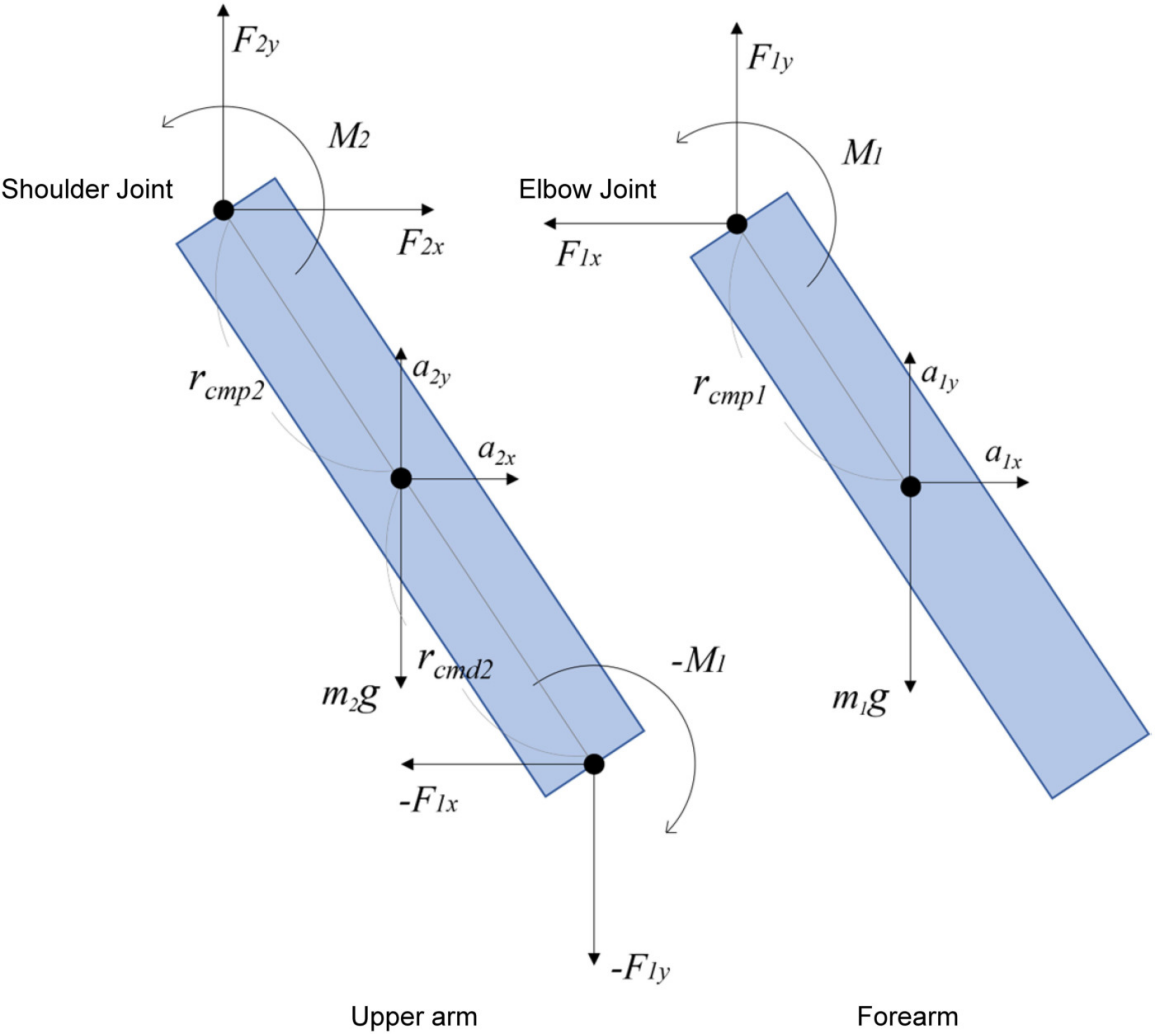
Schematic diagram of the free body diagram of the upper extremity: for illustrative purposes, the upper arm and forearm segments are depicted separately. Each joint is subjected to rotational moments and translational forces acting on the segment's center of mass. At the elbow joint, the reactive moment opposing *M_1_* is represented as –*M_1_*.

When we applied the above principles to movements on the whole plane of the body, we obtained the upper limb, comprised of two rigid bodies (upper arm and forearm) articulated at two hinge joints (shoulder joint and elbow joint), as shown in Figure 3. We modeled the upper limb and performed a two-dimensional analysis.

The pattern of the equations was the same for the two segments. Distal and proximal forces and moments of force and gravity were present in all segments.

The equation of motion of the elbow in the *x*-axis direction is

(3) $$F_{1x}m_{1}a_{1}x$$

from

$$\Sigma F_{x}=\;m_{1}a_{1}x$$ (4)

For the *y*-axis direction

$$F_{1y}+m_{1}g\;=\;m_{1}a_{1x}$$ (5)

where a_1x_ and a_1y_ are the acceleration, the displacement of the coordinates from one previous frame, and the sampling rate (fps).

For the moment, around the center of mass

$$\Sigma M=I_{1}a_{1f}$$ (6)

*I_1_* is the moment of inertia, and *I_1_ = m_1_ r_1_* is the moment of inertia.

*m_1_ and r_1_* are calculated by the body part inertia coefficient (Dempster).

From the above, the moment of the elbow joint *M_1_* is

$$M_{1}+r_{cmp1}\times F_{1x}+r_{cmp1}\times F_{1y}=\;I_{1}\alpha_{1}$$ (7)

Equation 1: Calculation of elbow joint moment (Nm)

The following is an example of a case in which the equation of motion in the *x*-axis direction, *ΣFx* *=* *m_２_ a_２x_* from the equation of motion in the *x*-axis direction

$$F_{2x}-\;F_{1x}=\;m_{2}a_{2x}$$ (8)

From the same equation of motion for the *y*-axis direction

$$F_{2y}+m_{2}gF_{1y}=m_{2}a_{2y}$$ (9)

For moments around the center of mass*,*

$$\Sigma M={I_{2a_{2f}}}_{f}$$ (10)

The shoulder joint moment from *M₂* is given by considering the reaction force due to the elbow joint calculated by Equation 1.

$$M_{2}+r_{cmp2}\times F_{2x}+r_{cmp2}\times F_{2y}+r_{cmd2}\times -F_{1x}+r_{cmd2}\times -F_{1y}-M_{1}=\;I_{2}\alpha_{2}$$ (11)

Equation 2: Calculation of shoulder joint moment (Nm)

**Figure 4:**
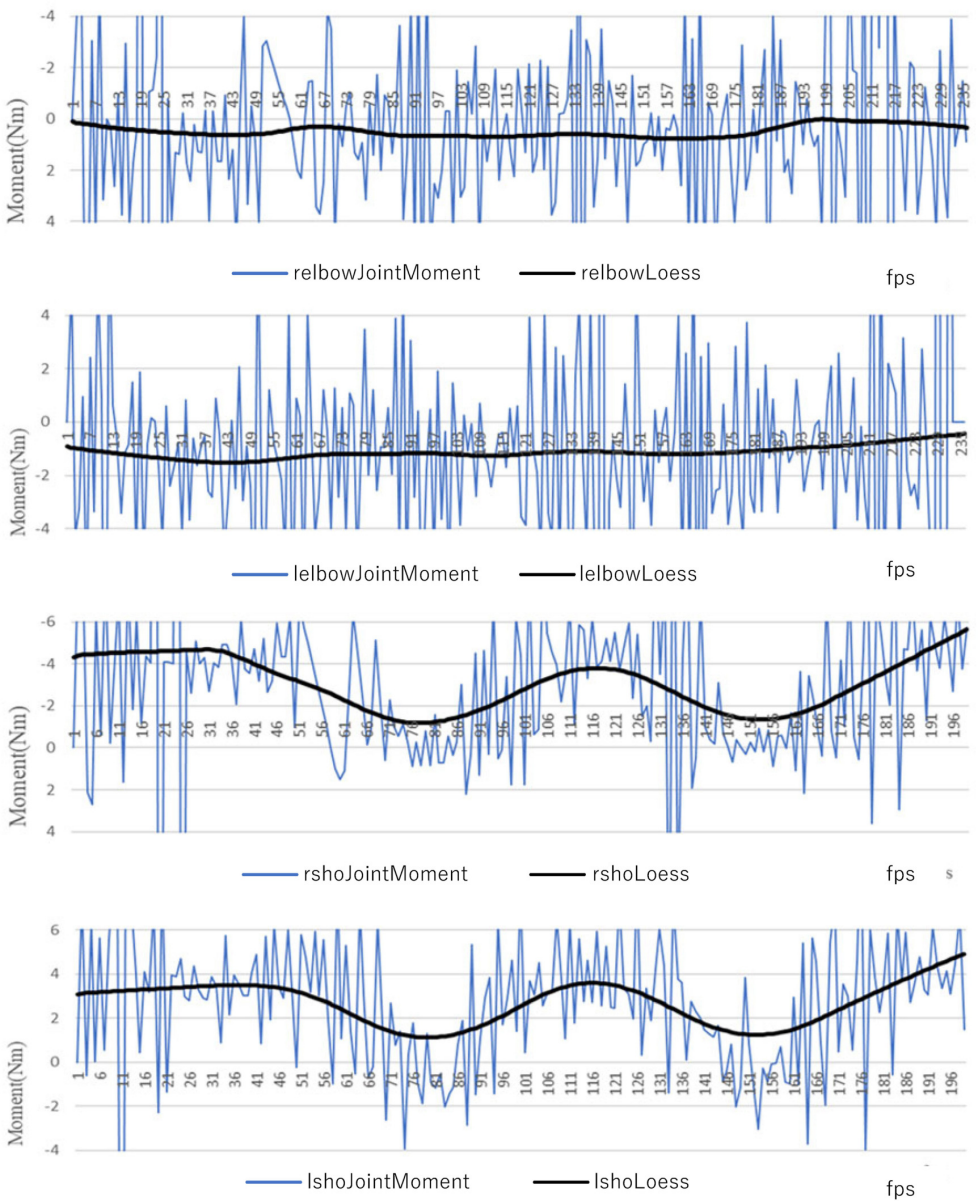
Shoulder joint moments in regular elderly participants: From top to bottom, moments estimation results of right elbow joint torque, left elbow joint torque, right shoulder joint torque, and left shoulder joint torque.

The analysis of healthy older adults yielded results consistent with those presented in Figure 4. The solid black lines in all figures indicate the smoothed moments. LOESS (16), a locally weighted nonparametric regression, was used for optimal smoothing. Ideally, the movement measured in this study should be a repetition of shoulder joint abduction with the elbow joint held in the flexed position simultaneously on both sides. In this study, we used an external Excel add-in (Pelitter technical services). The smoothing parameter was set as *α* = 0.15 for all data. Since this movement does not involve flexion and extension of the elbow joint, the change in elbow joint moment was minimal. Curves of the shoulder joints (left and right) illustrated symmetrical and stable movement and timing at the shoulder joint. The shoulder joints were controlled symmetrically and stably in motion and timing, which is considered ideal.

### 2.3 Motivation in exercise questionnaire (MEQ)

This study aimed to examine the effects of providing feedback on the motivation to exercise by administering a questionnaire. We assessed older people's willingness to exercise with our questionnaire, the MEQ (Motivation in Exercise Questionnaire), shown in Table 1. The maximum score for each question was 5 points, and participants were asked to score themselves on whether or not their thoughts applied to each question. Question 1 was designed to investigate the degree of willingness to participate in exercise at the time of the survey. Question 2 was formulated to examine the degree of understanding regarding improvement in one's exercise performance resulting from participation in exercise. Question 3 was aimed at investigating the degree of willingness to continue exercise. Standardized Cronbach's *α* values were 0.83 (initial intervention), 0.80 (4 weeks), and 0.73 (8 weeks).

**Table 1:** Subjects information.

Subjects group	Male	Female	Age mean	Age SD	Weight	Weight SD	Height	Height SD
Feedback	11	3	78.5	7.6	78.5	7.89	161.1	8.94
Control	7	10	80.7	8.01	80.7	8.25	155.1	7.95
	CVA	HV	DM	PD	(Male, Female)			
Feedback	6 (2,4)	2 (0,2)	3 (3,0)	3 (3,0)				
Control	6 (4,2)	3 (2,1)	5 (1,4)	3 (1,2)				

Table 1: Questionnaire seat of MEQ (Motivation in Exercise Questionnaire, 5-point rating)

1. Did participating in the exercise motivate you to participate in the exercise?
2. Were you able to understand changes in your athletic ability?
3. Do you want to continue exercising?

### 2.4 Research plan

This research hypothesizes that the sustained engagement in rehabilitation is significantly enhanced by the objective monitoring of motor skill progression and the subsequent provision of insightful feedback. To achieve this, our custom-developed program estimates upper limb joint moments by meticulously processing coordinates derived from advanced AI-based markerless motion capture technology. The primary objective of this investigation is to visually represent and deliver actionable feedback regarding participants' assessments of their motor function. This approach is designed to foster a more profound comprehension of evolving motor capabilities and, crucially, to amplify their motivation for consistent participation. Following the intervention, the efficacy of the feedback mechanism in promoting rehabilitation adherence will be evaluated through an original questionnaire.

The study involved 31 late-stage elderly patients receiving rehabilitation services at a hospital. To ensure a diverse representation of common geriatric conditions, participants were drawn from four distinct disease groups: cerebrovascular disease, Parkinson's disease, diabetes mellitus, and walking problems specifically associated with hallux valgus.

A non-randomized assignment design was employed to allocate participants into two groups: an intervention group that received feedback and a control group that did not. Care was taken to balance the number of participants from each disease category across both groups, aiming to minimize potential confounding effects from disease-specific characteristics. The design was referred to APA JARS (Journal Article Reporting Standards) - Quant - Module B (Reporting Standards for Studies Using Nonrandom Assignment) (https://apastyle.apa.org/jars) and APA Publication Manual 7th edition (17).

The intervention period spanned 8 weeks of structured exercise. Feedback was a key component for the intervention group, introduced after the initial 4 weeks of exercise. An additional 4-week follow-up period after the feedback phase allowed for the assessment of sustained effects.

**Figure 5:**
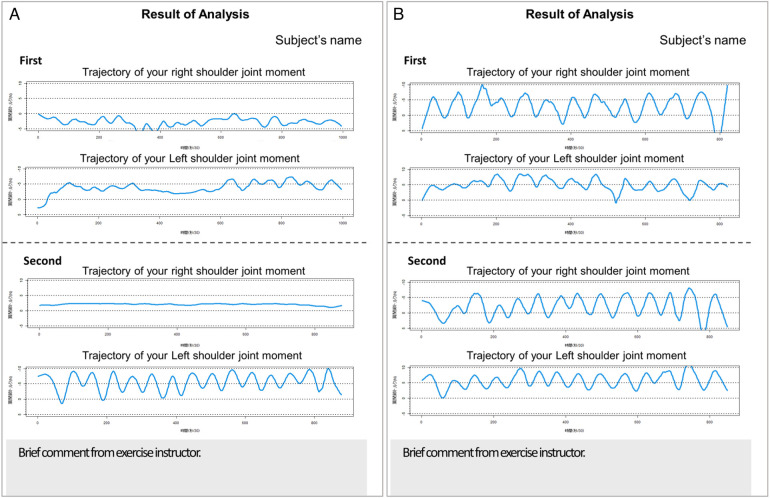
Examples of feedback presented to subjects: **(A)** An example of a participant who showed relatively improved shoulder joint control. **(B)** An example where improvement was observed, but asymmetry between the left and right sides remained.

To evaluate the impact of the intervention, MEQ was administered at the start of the exercise program, four weeks later, and four weeks after the follow-up period. To ensure fairness to the participants, feedback was given to the control group at the end of the survey period. This design ensured that all participants received feedback during the survey period. This scheme is illustrated in Figure 5.

The example of the feedback sheet is shown in Figure 5. The feedback sheet included the analysis results from the initial session and, one week later, in a format that allowed for comparison.

As illustrated by the two representative participants in Figure 5, outcomes varied across individuals, with some showing clear improvements and others exhibiting little to no change. It also included brief comments from the exercise instructor.

The subject group in this intervention did not have specialized knowledge about interpreting biomechanical analysis. Therefore, after the exercise, the instructor gave an oral explanation of the evaluation of each graph included in the sheet. Comparing the analysis results with those from the previous exercise, explanations were given about the graphs, such as “Is there a difference in the magnitude of the shoulder joint moment exerted?” and “Is the timing of the shoulder joint moment exerted on the left and right sides symmetrical?” Exercise instructors' comments were restricted to explaining outcomes, refraining from direct encouragement.

Changes in questionnaire scores were examined by multiple regression analysis with explanatory variables of the kind of disease and with or without feedback. Then, changes in questionnaire scores were analyzed using a paired t-test and a Wilcoxon signed-rank test.

#### 2.4.1 Subject and subject groups

##### 2.4.1.1 Participant characteristics

Participants were thirty-one patients prescribed exercise therapy by their physicians at a Rehabilitation Hospital. Participants included individuals with CVD cerebrovascular disease (*n* = 12), hallux valgus (*n* = 5), diabetes (*n* = 8), and Parkinson's disease (*n* = 6). These were the majority of the symptoms experienced by the clients of the hospital. Other restrictions, such as sex, age, height, and weight, were not applied to the participation. Self-selection of participants to the study did not occur.

Eighteen participants were male and 13 were female. Mean age of all participants was 79.70, SD = 8.18, 95%CIs[76.65, 82.75]. Mean height of all participants was 157.92 cm, SD = 9.23, 95%CIs[154.47, 161.36]. Mean weight of all participants was 60.16 kg, SD = 14.53, 95%CIs[54.73, 65.58].

##### 2.4.1.2 Assignment method

The experiment was done with a non-random assignment design. Participants were divided into two groups: with feedback (intervention group) and control group (without feedback), by balancing the number of participants for each disease. The division of groups was administered by the doctors, physical therapists, and exercise trainers.

The feedback group has 14 patients; there were 6 CVD, two hallux valgus, three diabetes and 3 Parkinson's disease patients. Males were 11 and females were 3. Mean age of feedback group was 78.50, SD = 7.89, 95%CIs[73.94, 83.05]. Mean height was 161.13, SD = 9.28, 95%CIs[155.77, 166.50]. Mean weight was 66.85, SD = 17.15, 95%CIs[56.95, 76.75].

The control group has 17 patients; there were 5 CVD, three hallux valgus, five diabetes, 3 Parkinson's disease, and one patient had a missing record in the questionnaire. Males were seven and females were 10. Mean age of the control group was 80.75, SD = 8.52, 95%CIs[76.20, 85.29]. Mean height was 155.10, SD = 8.47, 95%CIs[150.58, 159.61]. Mean weight was 54.29, SD = 8.66, 95%CIs[49.68, 58.90].

Further analyses were done based on the distinction of the two groups.

On masking, participants were not explicitly aware of condition assignments, because the two groups had their exercise separately.

#### 2.4.2 Feedback report

The objective of this study was to visualize participants' evaluations of their motor function and to offer constructive feedback. This approach aims to enhance their understanding of changes in motor function while fostering motivation to engage in exercise activities.

The feedback sheet presents the analysis results of the left and right shoulder joint rotation moments, showing the initial measurement and the results from the subsequent session four weeks later. Comments regarding the interpretation of the analysis results in the lower section include remarks on the stability, left-right symmetry, and smoothness of the shoulder joint rotation moment exertion. Figure 5 shows the results of two participants.

For the feedback group, the results were presented by professional trainers and a prosthetics and orthotics professional who regularly provides exercise guidance, explaining how to interpret the graphs to all participants. Subsequently, the staff distribute the individual feedback sheets and answer questions.

Considering the effect on answers on MEQ, comments from exercise instructors were limited to explanations of the results, and did not provide direct encouragement.

**Figure 6:**
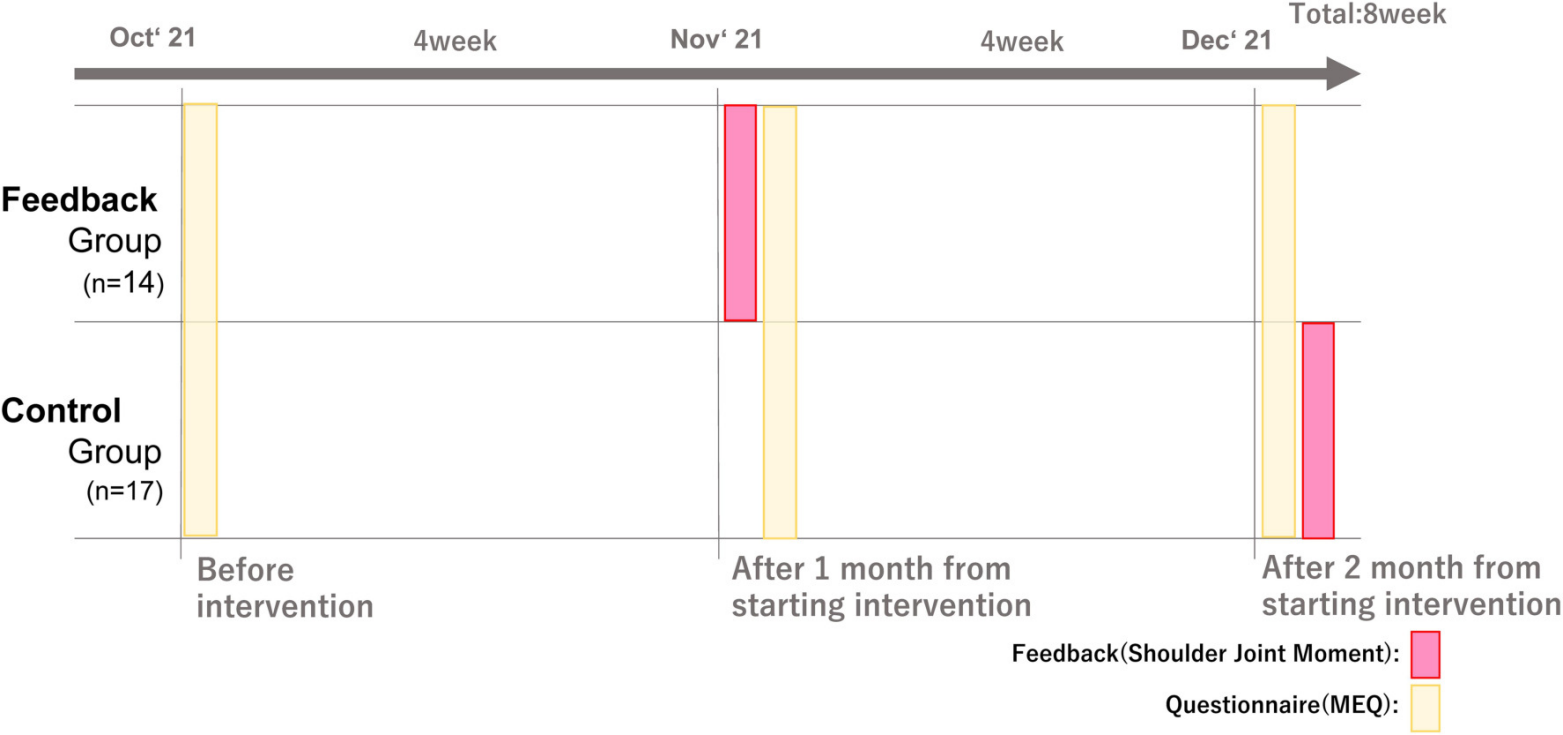
Research plan for validating the effectiveness of the feedback scheme.

As shown in the next section and in Figure 6, the control group also receives a feedback report at the end of the research period.

#### 2.4.3 Experiment design

##### 2.4.3.1 The timeline of the experiment

The intervention period was 8 weeks, including a 4-week feedback and a 4-week follow-up. The upper panel shows the schedule for the group that received feedback, while the lower panel shows the schedule for the control group. The feedback sheets administered to each group included a comparison of the analysis results at the time of the initial intervention and one week later. In Figure 6, MEQ surveys were administered at the time points highlighted in beige, and feedback was provided at the time points highlighted in red.

##### 2.4.3.2 Sample size and power analysis

In this research, the promoting effect of the feedback is examined with MEQ. MEQ was administered at the start of the exercise program, four weeks later, and four weeks after the follow-up period. Changes in MEQ scores at each group (the Feedback group and the control group) along with the time were examined.

A power analysis was conducted to determine the required sample size for a paired t-test, with the “effectsize” package (18) on R 4.5.0. The test type was set as “paired t-test” and “one-sided (greater)”. The significance level was set at 0.1, the desired power was 80% and the anticipated effect (Cohen's d) was 0.7 (medium-large effect). The script was pwr::pwr.t.test(+ sig.level = 0.1, + powe*r* *=* 0.8, + d = 0.7, + type = “paired”, + alternative = “greater”).

To detect this effect size, the estimated sample size was 10.12. In section 3.2, a paired t-test was conducted for the feedback group comprising 11 participants and the control group consisting of 14 participants. In both groups, the number of participants exceeded 10.12.

#### 2.4.4 Analysis method

The participant profile data included whether feedback was provided, age, sex, height, weight, and the disease being treated. The four disease groups included cerebrovascular disease (CVD), Parkinson's disease (PD), diabetes mellitus (DM), and hallux valgus (HV). To protect personal information, the names of the participants were not used in data processing or storage.

At first, the relations between MEQ scores and the variation of diseases were examined. Using a multiple regression analysis model, where the change in responses to the questions was the objective variable, and the profiles, including diseases, with and without feedback, were the explanatory variables.

In the following analysis, we examine changes in MEQ scores over four-week spans with a paired t-test and the Wilcoxon signed-rank test.

This study was approved by the Ethics Committee for Medical Research of Hiroshima International University (approval number: Ethics 21-017). The purpose of the study was fully explained to all participants, and written consent was obtained before the experiment.

## 3 Results

### 3.1 Participant flow

**Figure 7:**
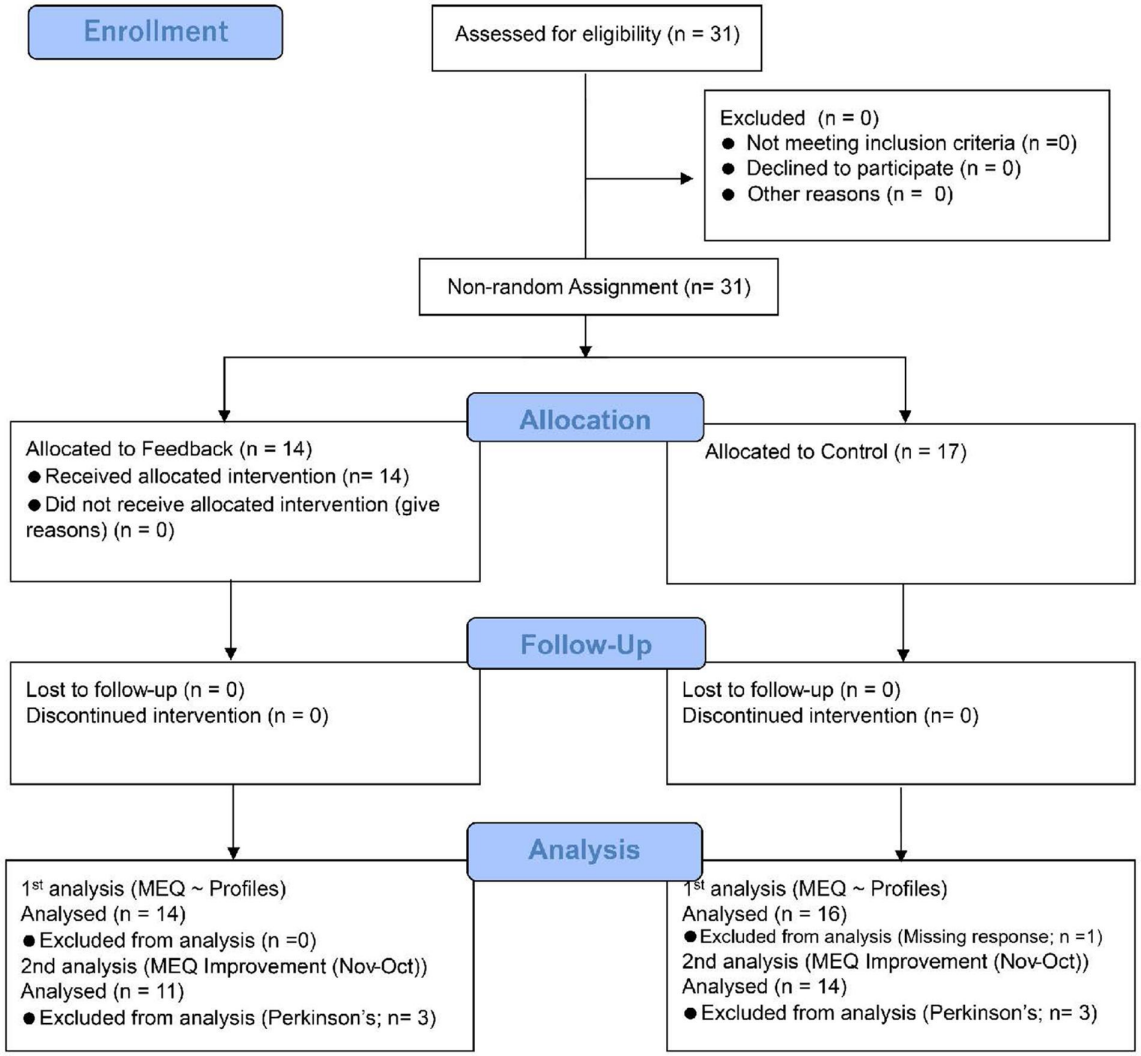
Participant flow diagram.

A total of 31 participants were assessed for eligibility between October and December 2021. The flow of participants through the trial is detailed in Figure 7. All 31 individuals assessed were eligible to participate. They were assigned to one of two groups: the feedback group (*n* = 14) or the control group (*n* = 17).

As noted in 2.4.1, the non-random assignment of participants to two groups was by balancing the number of participants for each disease. The assignment was administered by the doctors, physical therapists, and exercise trainers.

In the feedback group, all 14 participants were included until the follow-up and the first analysis, which examines MEQ scores and relations with profiles including diseases. In the second analysis on the improvement of MEQ scores along with the training, 3 Parkinson's disease participants were excluded. Then, *n* = 11 at the second analysis. The following section shows the details.

In the control group, all 17 participants were included until the follow-up. At the first analysis, one participant missed the question on disease, so the analysis was conducted on 16 participants. In the second analysis, 3 Parkinson's disease participants were excluded. Then, *n* = 14 at the second analysis.

### 3.2 Examination of the disease variations on the MEQ score

When investigating individuals' willingness to exercise following the feedback on analysis results, it is anticipated that interactions will emerge based on their profile data.

A multiple regression analysis on all participants (including both the Feedback and Control groups) was conducted to examine the effect of disease variation on the difference in MEQ-Q1 scores between October and November (from before the intervention to one month after the intervention started). The score difference was set as the dependent variable. The disease profiles and the presence or absence of feedback were included as independent variables.

The regression model is defined as Q1 (Nov-Oct)∼Diseases + Feedback + bias. The base of the estimated coefficient value (set as baseline 0) of the Diseases is hallux valgus (HV). The base of the Feedback is Yes (as 0), then, the absence of the Feedback was coded as 1. Q1 and other MEQ questions are coded as values of 1–5, the same as the rating of the questionnaire.

The *t*-test was performed on each partial regression coefficient. DOF is 25 (31 observations -6 explanatory variables with an intercept). The findings revealed that the partial regression coefficient for participants with Parkinson's disease was significantly negative compared to those in other disease groups (*t*(25)* =* −2.43, *p* = 0.022, 95%CI [−1.85, −0.15]) (Table 2). Given these results, the study focused on disease groups other than the Parkinson's disease group. Regression and *t*-test were computed with lm() of R4.5.0.

**Table 2:** Multiple regression coefficients on MEQ Q1 improvement from diseases and presence or absence of feedback.

Term	Estimate	Std error	*t* ratio	*p*	95% CI LL	95% CI UL
Intercept	0.169	0.228	0.74	0.464	−0.300	0.638
Deseases_Parkinson	−1.003	0.412	−2.43	0.022*	−1.851	−0.155
Deseases_CVD	−0.109	0.342	−0.32	0.754	−0.814	0.597
Deseases_Diabetes	0.414	0.375	1.10	0.281	−0.359	1.187
Feedback_No	−0.332	0.220	−1.51	0.143	−0.785	0.121

* Statistical significance at the 5% level (*p* < 0.05).

**Figure 8:**
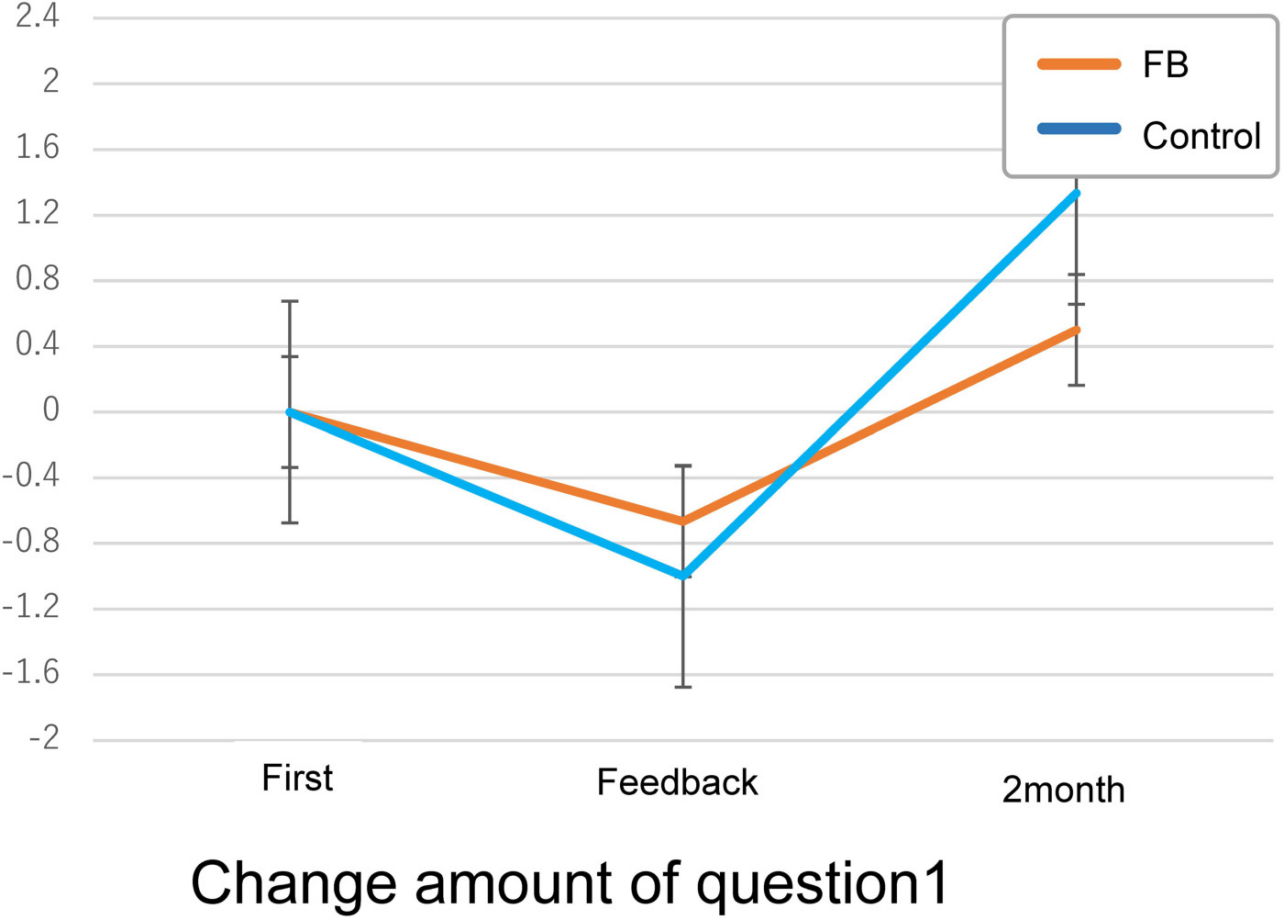
Parkinson's disease patients’ MEQ Q1 difference over 2 months.

Furthermore, Figure 8 shows the difference in Parkinson's disease patients' MEQ Q1 over two months. The orange line represents the trend in scores for the group that received feedback, while the blue line represents the trend for the group that did not. A score decline was observed regardless of the presence or absence of feedback. Both groups show decreased motivation after one month from the start, and two months later. Both groups show recovery of motivation.

The regression model's linearity and normality of distribution were verified using the Lack of Fit test. The Lack-of-Fit test statistically assesses whether the linear model provides an adequate fit to the data. The F-statistic is the ratio of the mean square for the residual error to the mean square for pure error (variation among any replicates). If the lack of fit is significant, this suggests that the model is not capturing the true underlying relationship. The null hypothesis was that there was no significant difference from a linear model. A significant *p*-value (typically < 0.05) indicates a lack of fit, suggesting that a more complex model (e.g., a curvilinear model) might be more appropriate. In this case, the null hypothesis was not rejected (*p* = 0.571) (Table 3), indicating acceptance of the linear regression model.

**Table 3:** Analysis of variance for lack of Fit of the linear regression model predicting MEQ Q1 improvement from diseases and presence or absence of feedback.

Source	SS	df	MS	*F*	*p*
Residual error					
Lack of Fit	3.015	3	1.005	0.685	0.571
Pure Error	32.300	22	1.468		
Total	35.315	25			

SS, Sum of Squares; df, degrees of freedom; MS, Mean Square. The total residual error is decomposed into the Lack of Fit and Pure Error components.

The regression model's fitting was also examined using the Rainbow test for linearity (19). The Rainbow test is that even if the true relationship is non-linear, a good linear fit can be achieved on a subsample in the “middle” of the data. The null hypothesis is rejected whenever the overall fit is significantly worse than the fit for the subsample. Computation of the Rainbow test was done with the raintest() function of the R 4.5.0's lmtest package. The null hypothesis was not rejected, indicating the acceptance of the model's linearity (Rain = 0.2652, df1 = 16, df2 = 9, *p*-value = 0.9899).

The homoscedasticity of the model was examined with the Studentized Breusch-Pagan test (20). It tests whether the variance of the errors from a regression is dependent on the values of the independent variables. The null hypothesis is rejected whenever significant heteroskedasticity is present. The bptest() function of lmstat was used. The null hypothesis was not rejected (BP = 10.42, df = 5, *p*-value = 0.064). The heteroskedasticity exists, but it is not statistically significant.

The Variance Inflation Factor examined the absence of multicollinearity. The VIF provides an index that measures how much the variance (the square of the estimate's standard deviation) of an estimated regression coefficient is increased because of collinearity. The smallest possible value of VIF is one (absence of multicollinearity). Generally, a VIF value exceeding 5 or 10 typically indicates a problematic level of collinearity (21). In categorical explanatory variables, Generalized VIF with degree-of-freedom GVIF^(1/(2*Df)) was appropriate. In this case, both the GVIF and the regulated VIFs were close to 1, indicating the absence of multicollinearity (Table 4). VIF was computed with vif() of car package of R4.5.0.

**Table 4:** Variance inflation factor of the linear regression model predicting MEQ Q1 improvement from diseases and presence or absence of feedback.

Effect	GVIF	df	GVIF^(1/(2*Df)
Diseases	1.051	4	1.006
Feedback	1.051	1	1.025

### 3.3 Repeated measures analysis using a mixed-effects model for the MEQ score

Before the detailed analysis of feedback, we have analyzed the comprehensive analysis of independent variables related to the effect of feedback, excluding cases of Parkinson's disease. Repeated measures analysis using a mixed-effects model was applied to explore relations between the MEQ score and participants’ background and control variables. MEQ Q1 score was set as the dependent variable. Between independent variables, the fixed effect variables are Diseases, with/without Feedback, Periods (1st: before intervention and 2nd: one month after), and Female/Male. The nested random variables were the Subject in the Female/Male group. The REML method of JMP 19.01 (JMP Statistical Discovery LLC.) was used for computation.

Table 5 shows the *F*-test table of the fixed effects. Feedback affected the MEQ (*p* = 0.084). Periods (1st or 2nd) slightly affected the MEQ (*p* = 0.164). Female or Male affected the MEQ (*p* = 0.108).

**Table 5:** F table on the fixed effects on repeated measures analysis using a mixed-effects model: MEQ Q1 score is the dependent variable. The fixed effect variables are Diseases, with/without Feedback, Periods, and Female/Male.

Effect	*N* of Parameters	df	df (denominator)	*F*	*p* (Prob > F)
Diseases	2	2	19	1.2798	0.3010
Feedback	1	1	19	3.3288	0.0838
1stOr2nd	1	1	23	2.0631	0.1644
Female/Male	1	1	19	2.8477	0.1079

There was no significant random effect on nested female/male participants, with *t* and *p* values.

The effect of gender differences on MEQ responses is not negligible; however, this study prioritized assigning participants to each of the different diseases within the experimental design. Since the variation of the Diseases was not significant (*p* = 0.30), we have not added the exclusion of particular diseases in subsequent analyses. Therefore, in the Feedback group, there were 6 participants with CVD, 3 with Diabetes, and 2 with Hallux Valgus; in the Control group, 5 with CVD, 5 with Diabetes, and 3 with Hallux Valgus.

### 3.4 The *t*-test for the MEQ score differences at the start and at the feedback point, among participants other than those with Parkinson'**s** disease

We have examined the feedback effect by comparing the differences in MEQ scores between October, November, and December in the feedback group and the control group. Distribution parameters of the MEQ score difference were examined as the repeatedly measured data on the same subject group, which underwent evaluations over four weeks.

Paired *t*-tests were conducted to determine whether there was any change between the two groups after four weeks, from October to November. As shown in Figure 6, the participants of the feedback group received their personalized report in November. MEQ was administered to both groups after the feedback group received the report.

We compared MEQ scores on patients with conditions other than Parkinson's disease, such as Hallux Valgus, CVD, and Diabetes. As shown in Figure 7, the comparison of the feedback report involves 25 participants, with the feedback group comprising 11 participants and the control group consisting of 14 participants. In both groups, the number of participants exceeded the estimated sample size by 10.12, according to power analysis.

Garren & McGann Osborne (22) and Garren & Davenport (23) examined the robustness of the *t*-test against the departure from the normal distribution. Simulation results show that the *t*-test exhibits robustness to skewness. When the sample size is small and the Kurtosis is too low, meaning a much flatter distribution compared to the normal distribution, the *t*-test tends to increase the type I error (false positive). They show a conservative criterion for low-kurtosis cases as smaller than 1.83 for a sample size of 20. MEQ Q1 and Q3 have larger kurtosis, with values of 4.18 and 9.28, respectively. Q2 has a slightly smaller kurtosis of 1.78. Then, we examine the paired *t*-test for Q1 and Q3. For Q2, both the Wilcoxon signed-rank test and the paired *t*-test were applied. Analysis in lines below was computed with t.test(), wilcox.test(), and the effectsize package on R4.5.0.

**Figure 9:**
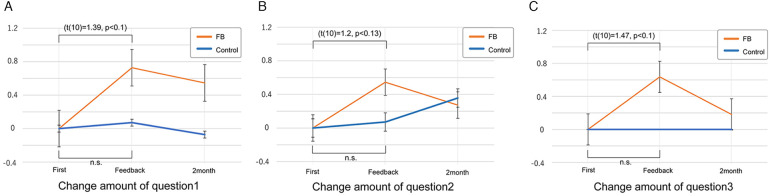
Graph of the difference in the score of MEQ: **(A)** shows the change in the score of Q1, “participating in the exercise motivates you to participate in the exercise?” **(B)** change in the Q2 score, “Were you able to understand changes in your athletic ability?" **(C)** Change in the Q3 score, “Do you want to continue exercising?”

Q1 scores motivation to the exercise, as “Did participating in the exercise motivate you to participate in the exercise?”, there was an increase in the score of the feedback group between the initial intervention and month 1 (one-sided: mean difference = 0.72, df = 10, t = 1.39, *p* = 0.09, 95%CI [−0.22, infinite (because of one-sided)], two-sided: *p* = 0.19, 95%CI [−0.43, 1.89]) (Figure 9A). Effect sizes are also computed. Three effect size measures show variation from 0.41 to 0.50. These measures are around “medium effect size (d = 0.5)” in Cohen (24), p.25–26) (Cohen's d = 0.50; 95%CI [−0.36, 1.34], Hedges' g = 0.48; 95%CI [−0.35, 1.29], Glass's delta = 0.41; 95%CI [−0.31, 1.12]).

During the first month, the control group that did not receive feedback had a tiny increase (one-sided: mean difference = 0.07, df = 13, t = 0.43, *p* = 0.33, 95%CI [−0.21, infinite], two-sided: *p* = 0.67, 95%CI[−0.28,−0.43]). The effect size is less than “small (d = 0.2)” in Cohen (ibid) [Cohen's d = 0.14; 95%CI (−0.61, 0.88)].

Although both groups show decreased scores after 2 months, the difference between the two groups remains almost the same.

For Q2, regarding the change in understanding of the improvement (“Were you able to understand changes in your athletic ability”), there was a non-significant increase in the score of the feedback group (*t*-test: one-sided: mean difference = 0.55, df = 10, t = 1.20, *p* = 0.13, 95%CI [−0.28, inf], two-sided: *p* = 0.26, 95%CI[−0.47,1.56], Wilcoxon Signed rank test: one-sided: V = 25.5, *p* = 0.15, two-sided: *p* = 0.30) (Figure 9B). The effect size measures show variation from 0.33 to 0.38. These measures are between “small effect (d = 0.2)” and “medium effect (d = 0.5)” (Cohen's d = 0.38; 95%CI [−0.47, 1.22], Hedges' g = 0.37; 95%CI [−0.45, 1.18], Glass's delta = 0.33; 95%CI [−0.42, 1.07]).

There was a tiny increase in the control group (one-sided: mean difference = 0.07, df = 13, t = 0.22, *p* = 0.42, 95%CI [−0.53, inf], two-sided: *p* = 0.84, 95%CI[−0.66,0.80]. Wilcoxon Signed rank test: one-sided: V = 14.5, *p* = 0.50, two-sided: *p* = 1.0). The effect size is less than “small” in Cohen (ibid) [Cohen's d = 0.06; 95%CI (−0.68, 0.80)].

Between months 1 and 2 after the intervention, the feedback group showed a decrease, while the group that did not receive feedback showed an increase, resulting in almost the same score in month 2.

For Q3, regarding motivation to continue exercising (“Do you want to continue exercising?”), the feedback group demonstrated an increase in score (one-sided: mean difference =0.64, df = 10, t = 1.47, *p* = 0.09, 95%CI[−0.14, inf], two-sided: *p* = 0.17, 95%CI[−0.33,1.59]) (Figure 9C). The effect size measures show variation from 0.34 to 0.44. These measures are in between “small effect” and “medium effect” (Cohen's d = 0.43; 95%CI [−0.42, 1.27], Hedges' g = 0.41; 95%CI [−0.41, 1.22], Glass's delta = 0.34; 95%CI [−0.35, 1.01]).

In contrast, the control group showed no difference in the mean difference values (0.0) between the two periods.

## 4 Discussion

### 4.1 Support of the original hypothesis

This study was conducted as an exploratory case study aimed at examining the effects of providing biomechanical analysis results to motivate participation in exercises.

The study was conducted following APA JARS standards using a non-random assignment design. Due to the practical constraints of experimenting with a hospital setting, the sample size was not large. Nevertheless, as indicated by the power analysis, it was possible to examine effects at a certain level. Furthermore, the effect size calculations demonstrated that there was at least a small effect, if not a medium effect.

Participants were thirty-one elderly individuals who were prescribed exercise therapy by their physicians. Participants included individuals with cerebrovascular disease, hallux valgus, diabetes, and Parkinson's disease. They attended a group exercise program once per week for approximately one hour.

They were divided into two groups: with feedback (intervention group) and without feedback (control group), by balancing the number of participants for each disease. The intervention period consisted of 8 weeks of exercise, with a feedback report provided after 4 weeks and a follow-up after an additional 4 weeks.

The original questionnaire (MEQ: Motivation in Exercise Questionnaire), comprising three questions, was used to assess motivation for exercising, self-understanding of improvements in athletic ability, and intention of continuing exercising. MEQ was administered at the start of the exercise program, four weeks later, and four weeks after the follow-up period.

Changes in questionnaire scores were examined by multiple regression analysis, with explanatory variables of the kind of disease and with or without feedback. The *t*-test was performed on each partial regression coefficient, and Parkinsonism participants had significantly negative values compared to those in other disease groups.

The subsequent analysis examined participants other than those with Parkinson's. The effect of feedback was analyzed using a paired *t*-test, and the results showed that the feedback group improved the level of motivation to participate in the exercise and motivation to continue exercising at 4 weeks from the beginning of the period. Improvements were not observed in the control group that did not receive the feedback. In the following 4 weeks, the feedback group showed a decrease in all scores.

The above results support the hypothesis that objective feedback reports on physical ability can increase motivation for exercise participation. As indicated by the changes in the scores of the questionnaire survey in this study, the participants' motivation to participate improved after the feedback was given; however, their motivation decreased 1 month after the feedback. Therefore, it is crucial to provide feedback as soon as possible. This study's physical function analysis system required approximately one cycle from measurement to analysis and feedback report. It was necessary to create a sheet for each participant, as the subjects participated in the exercise once a week. We believe that confirming the results of one's analysis during or immediately after participation in the exercise will reduce the differences between the analysis results and the participants' perceptions.

### 4.2 Interpretation

In this research, the number of participants was limited due to the clinical facility's capacity and care schedules. Assignments to the feedback and control groups were non-random, to balance the number of 4 diseases. These limitations resulted in relatively weaker effect sizes and minor statistical significance. In the subsequent research, we will enhance the motion capture and joint moment estimation workflow to enable conducting an RCT across multiple facilities.

### 4.3 Similarity of results

Oyake et al. (9) conducted a large-scale questionnaire study on motivating factors for rehabilitation among clinicians (physical therapists, occupational therapists, and speech-language-hearing therapists) and patients with neurological or orthopedic disorders. They found that the most frequently selected motivational factors were “realization of recovery” for patients and clinicians. Their questionnaire result accorded with our assumption.

Our study demonstrated that objective assessment of motor function can maintain and strengthen motivation to continue rehabilitation, as shown by changes over time in participants who participated in the same program. However, we also found that the effect diminishes after about one month, indicating the need for continuous feedback. Furthermore, we found that the effect may not be expected in some diseases (in our case, Parkinson's disease).

### 4.4 Generalizability

Objective feedback on physical ability is effective in motivating people to continue exercising, and this scheme is generally applicable to various diseases.

Although feedback was effective in other participants, it was also revealed that in Parkinson's disease patients, the desire to participate in exercises was reduced regardless of whether the feedback was present or not.

As exemplified by the results of the Parkinson's disease group in this experiment, feedback should be administered with caution to subjects with progressive and worsening diseases, such as amyotrophic lateral sclerosis (ALS) and spinocerebellar degeneration. In patients with progressive diseases, feedback may lead to a realization of the deterioration of their physical abilities, potentially resulting in counterproductive effects. These factors could become significant barriers to continued participation in physical activity and therefore require careful consideration. We are currently engaged in ongoing discussions with doctors, physical therapists, and exercise trainers to explore specific methodological approaches.

### 4.5 Implications

In this study, we experimented on the effect of implementing motor function assessment on participant motivation. Therefore, motor function assessments conducted in this study only showed changes in participants' upper limb joint moments and did not examine other motor functions in depth.

This research was an exploratory study, done with non-random assignment to balance the limited number of participants across four diseases. In further study, randomized assignment of a large number of participants over a long-term period will provide firm results. Furthermore, it is necessary to investigate changes in exercise motivation throughout long-term interventions and under conditions involving a broader range of disease groups.

In cases of progressive and serious illnesses, serious consideration must be given to what should be done.

## 5 Conclusions

We have developed our program to estimate upper limb joint moments using coordinates obtained through AI-based markerless motion capture. This study aimed to visualize and provide feedback on participants' assessments of their motor function to promote an understanding of changes in their motor function and to improve their motivation to participate in the exercise. Then, the promoting effect of the feedback is examined with a questionnaire. We observed an increase in motivation to continue exercise by providing an objective feedback report on motor function. Receiving continuous feedback more than once per month is necessary to maintain motivation for exercise.

## Data Availability

The original contributions presented in the study are included in the article/Supplementary Material, further inquiries can be directed to the corresponding authors.

## References

[1] Klein HA Jackson SM Street K Whitacre JC Klein G. Diabetes self-management education: miles to go. Nurs Res Pract. (2013) 2013:581012. 10.1155/2013/581012 23577243 PMC3616351

[2] Xing Y Yang S-D Wang M-M Feng Y-S Dong F Zhang F. The beneficial role of exercise training for myocardial infarction treatment in elderly. Front Physiol. (2020) 11:270. 10.3389/fphys.2020.00270 32390856 PMC7194188

[3] Mok A Khaw K-T Luben R Wareham N Brage S. Physical activity trajectories and mortality: population-based cohort study. Br Med J. (2019) 365:l2323. 10.1136/bmj.l2323 31243014 PMC6592407

[4] Yu F Vock DM Zhang L Salisbury D Nelson NW Chow LS Cognitive effects of aerobic exercise in Alzheimer’s disease: a pilot randomized controlled trial. J Alzheimers Dis. (2021) 80:233–44. 10.3233/jad-201100 33523004 PMC8075384

[5] Teasell R Mehta S Pereira S McIntyre A Janzen S Allen L Time to rethink long-term rehabilitation management of stroke patients. Top Stroke Rehabilitation. (2012) 19:457–62. 10.1310/tsr1906-457 23192711

[6] Sun F Norman IJ While AE. Physical activity in older people: a systematic review. BMC Public Health. (2013) 13:449. 10.1186/1471-2458-13-449 23648225 PMC3651278

[7] Shiraiwa K Ichii Y Murata S Abiko T Iwase H Naitou K 地域在住高齢者の行動変容ステージと身体機能の関係 (Relationships between the stage of change for exercise behavior and physical functions in elderly community residents). ヘルスプロモーション理学療法研究. (2017) 7:57–62. 10.9759/hppt.7.57 (in Japanese).

[8] Bandura A. The explanatory and predictive scope of self-efficacy theory. J Soc Clin Psychol. (1986) 4:359–73. 10.1521/jscp.1986.4.3.359

[9] Oyake K Yamauchi K Inoue S Sue K Ota H Ikuta J A multicenter explanatory survey of patients’ and clinicians’ perceptions of motivational factors in rehabilitation. Commun Med. (2023) 3:78. 10.1038/s43856-023-00308-7 37280319 PMC10244320

[10] Elendu C Amaechi DC Elendu TC Jingwa KA Okoye OK Okah MJ Ethical implications of AI and robotics in healthcare: a review. Medicine (Baltimore). (2023) 102:e36671. 10.1097/md.0000000000036671 38115340 PMC10727550

[11] Drazan JF Phillips WT Seethapathi N Hullfish TJ Baxter JR. Moving outside the lab: markerless motion capture accurately quantifies sagittal plane kinematics during the vertical jump. J Biomech. (2021) 125:110547. 10.1016/j.jbiomech.2021.110547 34175570 PMC8640714

[12] Cao Z Hidalgo G Simon T Wei S-E Sheikh Y. OpenPose: Realtime Multi-Person 2D Pose Estimation using Part Affinity Fields. *arXiv* [Preprint] (2018). 10.48550/arxiv.1812.08008 31331883

[13] Nakano N Sakura T Ueda K Omura L Kimura A Iino Y Evaluation of 3D markerless motion capture accuracy using OpenPose with multiple video cameras. Front Sports Act Living. (2020) 2:50. 10.3389/fspor.2020.00050 33345042 PMC7739760

[14] Winter DA. Muscle mechanics (Chapter 9); Kinetics: forces and moments of forcep (Chapter 5). In: Winter DA, editor. Biomechanics and Motor Control of Human Movement. 4th ed. Hoboken, NJ: John Wiley & Sons (2009). p. 224–49; 107–38. 10.1002/9780470549148.ch9

[15] Robertson DGE Caldwell GE Hamill J Kamen G Whittlesey SN. Research Methods in Biomechanics. 2nd ed. Champaign, IL: Human Kinetics (2014). Available online at: https://us.humankinetics.com/products/research-methods-in-biomechanics-2nd-edition

[16] Cleveland WS. Robust locally weighted regression and smoothing scatterplots. J Am Stat Assoc. (1979) 74(368):829–36. 10.1080/01621459.1979.10481038

[17] American Psychological Association. Publication Manual of the American Psychological Association: The Official Guide to APA Style. 7th ed. Washington, DC: American Psychological Association (2020).

[18] Ben-Shachar M Lüdecke D Makowski D. Effect size: estimation of effect size indices and standardized parameters. J Open Source Softw. (2020) 5(56):2815. 10.21105/joss.02815

[19] Utts JM. The rainbow test for lack of fit in regression. Commun Stat Theory Methods. (1982) 11:2801–15. 10.1080/03610928208828423

[20] Breusch TS Pagan AR. A simple test for heteroskedasticity and random coefficient variation. Econometrica. (1979) 47(5):1287–94. 10.2307/1911963 JSTOR 1911963. MR 0545960.

[21] James G Witten D Hastie T Tibshirani R. An Introduction to Statistical Learning: With Applications in R. New York, NY: Springer (2014).

[22] Garren ST McGann Osborne K. Robustness of T-test based on skewness and kurtosis. J Adv Math Comput Sci. (2021) 36(2):102–10. 10.9734/jamcs/2021/v36i230342

[23] Garren ST Davenport GH. Using kurtosis for selecting one-sample T-test or Wilcoxon signed-rank test. Curr J Appl Sci Technol. (2022) 41(18):46–55. 10.9734/cjast/2022/v41i1831737

[24] Cohen J. Statistical Power Analysis for the Behavioral Sciences. 2nd ed. Hillsdale, NJ: Lawrence Erlbaum Associates (1988).

[25] Bassuk SS Manson JE. Epidemiological evidence for the role of physical activity in reducing risk of type 2 diabetes and cardiovascular disease. J. Appl. Physiol. (2005) 99:1193–204. 10.1152/japplphysiol.00160.2005 16103522

[26] Matsuura K Ishihara S Matsuura A Morinaga K. Rehabilitation exercise aid system with biomechanical analysis and AI image recognition. In: Nojima Y Kim Y-T, editors. Proceedings of the Joint 10th International Conference on Soft Computing and Intelligent Systems and 19th International Symposium on Advanced Intelligent Systems (SCIS&ISIS 2018) in conjunction with Intelligent Systems Workshop 2018 (ISWS 2018). Toyama, Piscataway, NJ: IEEE (2018). p. 57–61.

